# Development of temporary subtropical wetlands induces higher gas production

**DOI:** 10.3389/fmicb.2013.00056

**Published:** 2013-03-15

**Authors:** Eliete B. Canterle, David da Motta Marques, Lúcia R. Rodrigues

**Affiliations:** Laboratório de Ecotecnologia e Limnologia, Instituto de Pesquisas Hidráulicas, Universidade Federal do Rio Grande do SulPorto Alegre, Brazil

**Keywords:** ecosystem development, % carbon, % organic matter, CH_4_, CO_2_

## Abstract

Temporary wetlands are short-term alternative ecosystems formed by flooding for irrigation of areas used for rice farming. The goal of this study is to describe the development cycle of rice fields as temporary wetlands in southern Brazil, evaluating how this process affect the gas production (CH_4_ and CO_2_) in soil with difference % carbon and organic matter content. Two areas adjacent to Lake Mangueira in southern Brazil were used during a rice-farming cycle. One area had soil containing 1.1% carbon and 2.4% organic matter, and the second area had soil with 2.4% carbon and 4.4% organic matter. The mean rates of gas production were 0.04 ± 0.02 mg CH_4_ m^−2^ d^−1^ and 1.18 ± 0.30 mg CO_2_ m^−2^ d^−1^ in the soil area with the lower carbon content, and 0.02 ± 0.03 mg CH_4_ m^−2^ d^−1^ and 1.38 ± 0.41 mg CO_2_ m^−2^ d^−1^ in the soil area with higher carbon content. Our results showed that mean rates of CO_2_ production were higher than those of CH_4_ in both areas. No statistically significant difference was observed for production of CH_4_ considering different periods and sites. For carbon dioxide (CO_2_), however, a *Two-Way* ANOVA showed statistically significant difference (*p* = 0.05) considering sampling time, but no difference between areas. The results obtained suggest that the carbon and organic matter contents in the soil of irrigated rice cultivation areas may have been used in different ways by soil microorganisms, leading to variations in CH_4_ and CO_2_ production.

## Introduction

Land use and land-use changes in agricultural and forest systems, industrial development, and urban expansion are among the sources of the current anthropogenic emissions of greenhouse gases (GHG) such as carbon dioxide (CO_2_) and methane (CH_4_). These activities have contributed to change the carbon and nitrogen cycles in terrestrial and aquatic ecosystems. Particularly, the contribution of agricultural soils to CO_2_ and CH_4_ emissions depends on biophysical processes and on the incorporation and decomposition of organic residues in the soil (Muñoz et al., 2010).

Wetlands, as well as rice paddies, contribute between 15 and 45% of global methane emissions (Segers, 1998). Organic matter tends to accumulate in the sediments of wetland soils. The carbon storage in soil organic matter is due to suppressed decomposition rates resulting from long-term soil saturation with water (Bohn et al., 2007). Soil organic matter is composed of a complex mixture of decayed plant and soil matter, with polymeric arrangements of these materials with other organic substrates (Wilson et al., 1983), whose rate and extent of degradation under either oxic and anoxic conditions are dependent on many biologic controls, including soil organic matter quality (Baldock and Skjemstad, 2000; Kristensen and Holmer, 2001; Blodau, 2002; Keller and Bridgham, 2007; Österreicher-Cunha et al., 2012).

The submerged conditions in rice paddies and the rice plants, especially their roots, supply the soil with organic carbon compounds which are mineralized by microorganisms (Hogberg and Read, 2006). The relationship between rice plants and microorganisms is important because the available substrates regulate many processes related to the emission or removal of gases (Insam and Wett, 2008).

Carbon gas production from wetland soil depends upon the rate of carbon deposition and the rate of mineralization (Smith et al., 2003). The rate of mineralization depends on a series of both biological and environmental factors and their interactions (Kristensen and Holmer, 2001). While the wetlands methane emissions result from anaerobic decomposition processes in deeper layers of wetland soil, CO_2_ emissions are related to oxidation of methane in upper oxic soil layers and respiration (Bohn et al., 2007). The rice-paddy sediment provides the ideal conditions for methanogenesis (Roehm, 2005), because the sediment undergoes oxygen depletion due to high moisture and relatively high organic-substrate levels and the presence of a methane-producing subsurface anaerobic zone and an aerobic surface zone that oxidizes this gas (Whalen, 2005). Anaerobic mineralization of carbon is the principal regulator of methane production in the sediment (Segers and Kengen, 1998), where the activity of methanogenic microorganisms converts a relatively narrow layer of simple substrates to methane (Zinder, 1998). Hence, the methanogenic microbial community in rice field soil contributes about 13% to the global budget of CH_4_ (Lelieveld et al., 1998). Other gases such as CO_2_ are also related to microbial activity.

Gas emissions from wetlands normally show wide seasonal and temporal fluctuations, resulting from variations in environmental variables that regulate the microbial processes involved in the flux (Liikanen et al., 2006). The main local controls of CO_2_ productions from wetlands include the quality of soil organic substrates (Updegraff et al., 1995). Given the diverse variables that control the emission of methane and other gases from wetland environments (Yang and Chang, 1998; van der Nat and Middelburg, 2000; Conrad, 2002; Hirota et al., 2004; Whalen, 2005; Liikanen et al., 2006; Cheng et al., 2007; Welsch and Yavitt, 2007; Kao-Kniffin et al., 2010; Khosa et al., 2010; Inubushi et al., 2011), evaluating these processes and the effects of participating microorganisms is a complex task.

Rice fields are temporary wetlands formed by the extensive irrigation of areas for periods of approximately 90 days, with long postproduction drainage periods. Available data concerning these temporary wetlands at latitudes above 30° in the Southern hemisphere are sparse (Canterle et al., 2010; Rodrigues et al., 2011). Nevertheless, farming of irrigated rice represents the main crop for extensive lowland areas in southern Brazil, turning the soil of these areas in organic matter reservoirs, as the result of plant biomass accumulation at each production cycle. Considering that induced differences in the soil properties and microbiota characteristics may result in significant changes in the mineralization process, could rice fields, with different soil organic content and carbon, present different mineralization rates as measured by gas production? Therefore, the goal of this study is to describe the development cycle of rice fields as temporary wetlands, analyzing the dynamic of limnological variables and the mineralization process in the gas production (CH_4_ and CO_2_) in areas with different incorporated organic matter and carbon content in southern Brazil.

## Materials and methods

### Study site

**Figure 1:**
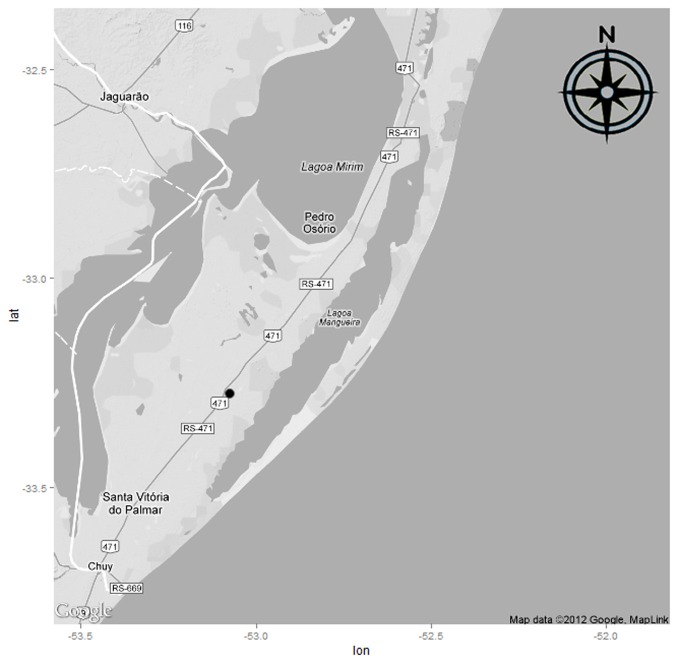
**Mangueira Lake, a large coastal lake in the state of Rio Grande do Sul, Southern Brazil, and localization of rice fields sampled (•)**.

The study was conducted during the 2005/2006 crop cycle in two areas of flooded-rice cultivation (BR-IRGA 410 cultivar). The water used to irrigate these areas was taken from Lake Mangueira (Figure 1) which, together with its contributing basin, form the Mangueira subsystem and cover an area of 1597 km^2^. This subsystem belongs to the Taim Hydrological System and is part of a gradient of floodplains characterized by the presence of freshwater wetlands and associated lakes, situated in Rio Grande do Sul State in southern Brazil (Motta-Marques et al., 2002). Part of the water used during irrigation of the cultivated areas returns to the lake via a drainage channel.

Each area has a soil with different organic-matter and carbon contents. Area 1 (S 33.288560°; W 53.092518°), 4 ha in extent, had soils with lower organic-matter and carbon contents. Area 2 (S 33.287297°; W 53.088699°), with an area of 1 ha, had higher organic-matter and carbon contents.

### Sampling and sample analysis

Sampling was conducted between 16/12/2005 and 16/03/2006, corresponding to the rice crop cycle between the period of flooding (first week of December) and harvest (last week of March). Five sample series for each area were taken in order to estimate the production of gases and bacterial abundance (BA), and four sample series (except in the last sampling) were taken for limnological analyses.

Water samples were collected from the water/sediment interface at each sampling site for the limnological analysis. Water temperature, dissolved oxygen and pH were determined using a multiparameter probe (Yellow Springs Instruments model YSI 6920). Ten 1-L water samples were collected for chemical analysis and chlorophyll a. The variables were determined as follows: total phosphorous (TP), soluble reactive phosphorus (SRP), total nitrogen (TN), and nitrate (NO3) were analyzed according to Mackereth et al. (1989). Total solids (TS) were estimated according to APHA (1999), and soluble reactive silicon (SRSi) was measured by the photometric method, using a commercial kit (Si Merck Spectroquant7 kit for silicate-sulfuric acid). Dissolved organic matter (DOM) was analyzed by the spectrophotometric method (Strome and Miller, 1978). Carbon (dissolved organic, DOC; and dissolved inorganic, DIC) was analyzed using a total organic carbon (TOC) analyzer (Shimadzu VCPH). Chlorophyll a was extracted from GF/F filters in 90% ethanol (Jespersen and Christoffersen, 1987) and measured by the spectrophotometric method (APHA, 1999).

Sediment samples from each area were collected during the first sampling (December/05) for chemical, element and macronutrient analyses as the following methods: percentage of organic matter was determinate by humid digestion, clay was determinate by densimeter method, K and P were determinates by Mehlich I method, S-SO_4_ extracted with CaHPO_4_ 500 mg L^−1^ of P, Zn and Cu were extracted with HCl 0,1 mol L^−1^, B was extracted with hot water, Mn exchangeable extracted with KCl 1 mol L^−1^ and % soil carbon was analyzed using a TOC analyzer (Shimadzu VCPH).

For the CH_4_ and CO_2_ analysis, at five sites within each area, the surface layer (about 10 cm) of the sediment was collected in PVC tubes, placed in plastic bags and stored in a refrigerator for no more than 2 days until incubation. At the sediment sampling sites, water samples from the water/sediment interface were collected in 50-mL flasks and also stored in the refrigerator until incubation was performed. In the laboratory, each sediment sample was homogenized and an aliquot of 6 g was placed in a 25-mL glass flask and mixed with 5 mL of the water collected at the same site, forming five sludge slurry samples per area. Prior to incubation, gaseous nitrogen (N_2_) was bubbled through the sludge slurry to create an anoxic environment (Minello, 2004). The flasks were closed with rubber septa, sealed with metal rings and incubated in the dark at a controlled temperature (20 ± 0.5°C). Gas production in the sediment was determined from the accumulation of CH_4_ and CO_2_ in the headspace of the incubation flasks (Casper, 1992). After a 5-d incubation period, an aliquot of the internal atmosphere of each flask was extracted to determine the CH_4_ and CO_2_ produced, using gas chromatography (Varian Star 3400, Varian) with a flame ionization detector, injector temperature of 120°C, a Chromosorb 102 column (12′ × 1/8″), (80/100) at 27°C, and using helium as the carrier gas. Gas production, measured by gas chromatography, was expressed as g^−1^ · L^−1^. The data conversions were carried out using the mass × sediment volume relation (obtained from the PVC cores used in the removal of sediment samples and volume of the sediment). We assumed a fixed sediment depth for gas production considering values observed in the literature. Data conversion to m^2^ was done considering the core sampling area (28.27 cm^2^) and assuming that most of the gas production was related to the upper 10 cm of the sediment layer (Casper, 1992).

For bacterioplankton abundance analysis, water samples from the water-sediment interface at each sampling point were fixed in 40% formaldehyde (final concentration in the sample = 4%) and prefiltered on quantitative paper (MN 640 d Macherey–Nagel; mean particle retention size 2.0–4.0 μm). Next, 2 ml of each sample was filtered and stained with acridine orange, and cells were concentrated onto 0.2-μm black polycarbonate membranes (GE), according to the modified protocol of Hobbie et al. (1977). BA, determined as cells per mL, was estimated using an inverted epifluorescence microscope (Olympus IX70).

### Data analysis

Data analysis was performed by using a *Two-Way* ANOVA to compare gas production, BA and limnological variables. With this approach the two sites were compared after extracting the temporal effect, at the same time as sampling period were compared with no effect of sampling site. Associations between the different variables (gases, limnological variables and BA at the water/sediment interface) were assessed through correlation analyses using Spearman correlation coefficients. Statistical analyses were done using SigmaPlot 10.0 software. A probability level of α = 0.05 was adopted to determine statistical significance.

## Results

Analysis of soil from the sampling sites showed that the organic-matter, carbon and clay contents and pH were all higher in Area 2 than in Area 1 (Table 1). The percentages of organic matter and carbon in Area 1 sediment were approximately half those of Area 2. The mean pH values were also higher in Area 2. Regarding macronutrients, Area 1 showed a higher mean value for phosphorus, while higher mean values were obtained for potassium and manganese in Area 2.

**Table 1:** **Results of chemical analyses of soil from the sampling sites**.

**Variable**	**Area 1**	**Area 2**
Organic matter (%)	2.4 ± 0.2	4.4 ± 0.2
Carbon (%)	1.1 ± 0.2	2.4 ± 0.6
Clay (%)	12.3 ± 4.0	15.7 ± 3.1
K (%)	148.7 ± 60.4	150.5 ± 29.1
P (%)	25.3 ± 10.8	10.6 ± 2.1
Ca/Mg	3.1 ± 0.2	4.7 ± 2.0
Ca/K	6.6 ± 1.6	31 ± 13.2
Mg/K	2.1 ± 0.4	6.7 ± 1.5
S (%)	9.5 ± 2.7	9.8 ± 2.9
Zn (%)	2.3 ± 1.2	2.3 ± 0.5
Cu (%)	1.3 ± 0.3	0.7 ± 0.0
B (%)	0.4 ± 0.1	0.5 ± 0.1
Mn (%)	50.7 ± 22.0	6.3 ± 0.6
pH	5.6 ± 0.2	6.2 ± 0.1

The mean values for the limnological variables in both areas are presented in Table 2. During the study period, both rice fields presented similar temperature and pH. Mean concentrations of dissolved oxygen were higher in Area 1 than in Area 2. The concentrations of nutrients and chlorophyll *a* showed a tendency to decrease over the course of the rice-crop cycle.

**Table 2:** **Results of analyses of water from the water/sediment interface samples**.

**Variable**	**Sampling 1**		**Sampling 2**		**Sampling 3**		**Sampling 4**	
	**Area 1**	**Area 2**	**Area 1**	**Area 2**	**Area 1**	**Area 2**	**Area 1**	**Area 2**
DIC (mg · L^−1^)	19.66 ± 4.4	24.79 ± 4.5	21.82 ± 2.3	28.32 ± 3.5	25.47 ± 2.5	26.38 ± 1.1	27.74 ± 2.8	29.19 ± 3.2
DOC (mg · L^−1^)	15.11 ± 8.6	18.33 ± 10.6	12.52 ± 5.9	10.16 ± 2.7	7.12 ± 1.7	6.33 ± 0.8	8.30 ± 1.7	7.42 ± 1.9
Total Solids (mg · L^−1^)	310 ± 102	274 ± 96	238 ± 20	247 ± 12	221 ± 10	211 ± 11	201 ± 61	201 ± 17
SRSi (mg · L^−1^)	3.22 ± 0.77	3.47 ± 0.68	1.03 ± 0.90	1.56 ± 1.04	1.76 ± 0.84	2.14 ± 0.48	0.36 ± 0.33	0.27 ± 0.09
DOM—UV_DOC_ (254 nm)	0.44 ± 0.26	0.30 ± 0.13	0.03 ± 0.03	0.05 ± 0.03	0.14 ± 0.03	0.18 ± 0.03	0.02 ± 0.01	0.01 ± 0.01
Chlorophyll *a* (μg · L^−1^)	29.33 ± 21.85	33.19 ± 22.80	39.47 ± 17.28	42.73 ± 21.35	2.51 ± 1.38	2.52 ± 0.64	2.25 ± 1.20	1.64 ± 0.66
TP (μg · L^−1^)	5.9 ± 4.5	3.2 ± 1.9	2.3 ± 1.5	2.8 ± 1.7	1.0 ± 0.3	1.5 ± 0.2	0.1 ± 0.0	0.1 ± 0.0
SRP (μg · L^−1^)	2.82 ± 2.31	1.21 ± 0.88	0.30 ± 0.38	2.25 ± 2.40	0.14 ± 0.03	0.18 ± 0.03	0.03 ± 0.02	0.02 ± 0.01
TN (μg · L^−1^)	100 ± 20	170 ± 70	40 ± 20	40 ± 10	30 ± 20	20 ± 10	20 ± 10	10 ± 5
NO_3_-N (μg · L^−1^)	50 ± 40	50 ± 20	20 ± 10	30 ± 20	10 ± 5	10 ± 5	NQ	NQ
Dissolved oxygen (mg · L^−1^)	7.6	5.9	10.6	6.8	9.9	7.7	5.9	5.5
Water temperature (°C)	22.1	21.2	27.2	26	23.8	22.8	26.3	28.4
pH	7.8 ± 0.2	7.4 ± 0.4	7.3 ± 0.3	7.2 ± 0.1	6.7 ± 0.4	6.9 ± 0.2	7.6 ± 0.4	7.3 ± 0.4

DOM, dissolved organic matter; DIC, dissolved inorganic carbon; DOC, dissolved organic carbon; SRSi, soluble reactive silicate; TP, total phosphorus; SRP, soluble reactive phosphorus; TN, total nitrogen; NO_3_, nitrate.

No statistically significant difference was observed for limnological variables between the areas. However, *Two-Way* ANOVA showed significant difference considering different periods in sites for DOC (*p* = 0.028); TS (*p* = 0.022); SRSi (*p* = 0.002); organic matter (UV-DOC 254 nm) (*p* = 0.024); chlorophyll *a* (*p* = 0.000) and NO_3_ (*p* = 0.020).

**Figure 2:**
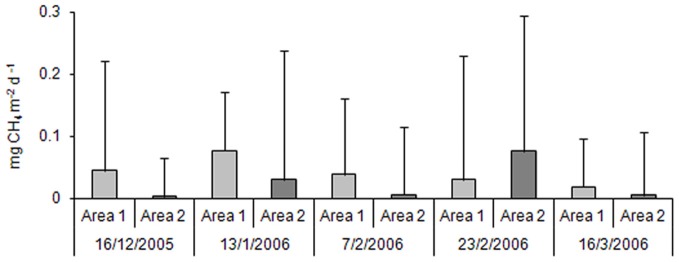
**Methane productions in the Area 1 and in the Area 2 throughout the rice crop cycle (*n* = 3)**.

No statistically significant difference was observed for production of CH_4_ considering different periods and sites. Methane production (Figure 2) showed a general decreasing gradient during the rice-crop cycle for the samples from Area 1; however, the samples from Area 2 showed irregular production levels, once that was observed alternating higher or lower values for methane production over time, although higher production values have been registered in all samples (see standard deviation). The highest values were obtained in Area 1 in the second sampling, after the application of urea; in Area 2, the effect of application of the inorganic fertilizer probably resulted in higher values only in the third sampling.

**Figure 3:**
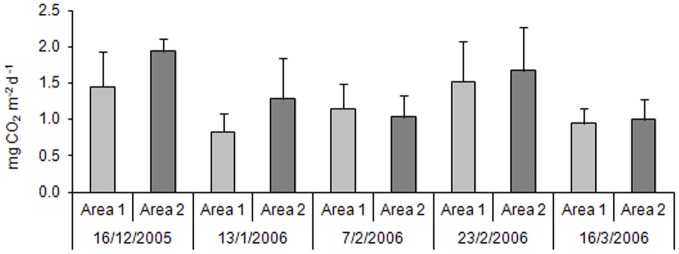
**Carbon dioxide productions in the Area 1 and in the Area 2 throughout the rice crop cycle (*n* = 3)**.

For CO_2_, the highest values were observed in the third sampling, in both areas (Figure 3). A *Two-Way* ANOVA showed statistically significant difference (*p* = 0.05) considering sampling time, but no difference between areas.

**Figure 4:**
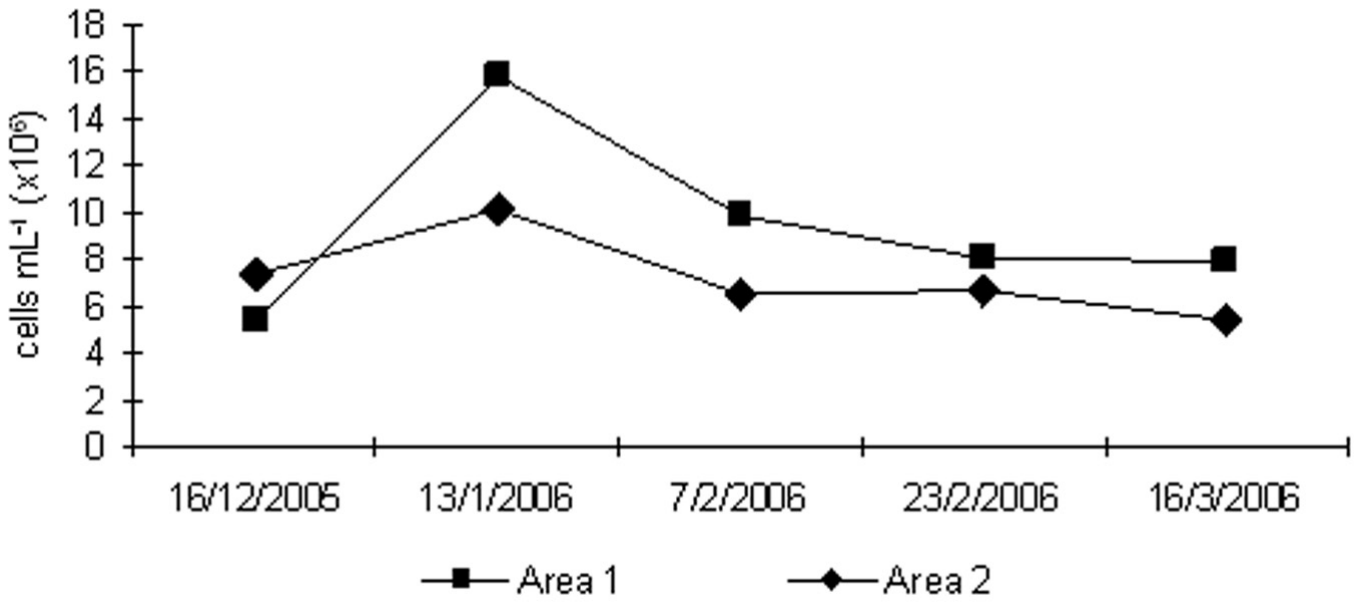
**Bacterioplankton abundance rates obtained from the water/sediment interface in both areas during rice cultivation**.

Although the bacteria present in the sediment samples from the sampling sites, as well the attached bacteria have not been analyzed, unpublished data of the authors obtained in another study show an increase in density of methanogenic and methanotrophs microbial groups in experiments carried out with samples of the sediment-water interface. In addition, samples from the sediment-water interface site presents, normally, a consortium of microbes; it was added to the sludge slurry incubated in the experiment. These facts could be used to infer the effect of the microbial groups from the water/sediment interface samples in the gas production at the upper layer of sediment. Bacterioplankton abundance rates in the two areas showed similar trends as the values obtained for CH_4_ and CO_2_ production, with higher abundances in Area 1, which showed lower soil % organic matter and % carbon (Figure 4). The highest values for BA were observed in the second sampling, after the application of inorganic fertilizer in both areas. A *Two-Way* ANOVA showed no statistically significant difference was observed for BA considering different periods and sites.

The correlation analysis of CH_4_ and CO_2_ production showed that gases in Area 1 were positively correlated with BA from the water/sediment interface samplings (CH_4:_ *r* = 0.54; CO_2_: *r* = 0.56; *p* < 0.05). On the other hand, in Area 2 the CH_4_ production was not correlated with soil components or BA from the water/sediment interface samplings, while CO_2_ production was negatively correlated (*r* = −0.60; *p* < 0.05) with BA from the water/sediment interface.

From the data obtained in this study, it is possible to estimate the contribution of temporary subtropical wetlands in the region, regarding the production of CH_4_ and CO_2_ gases. Considering the mean rates of gases production in the two areas (*n* = 5), mean methane production was 0.041 ± 0.02 mg m^−2^ d^−1^ in Area 1 and 0.024 ± 0.03 mg m^−2^ d^−1^ in Area 2, while the mean production rate for CO_2_ production presented 1.178 ± 0.3 mg m^−2^ d^−1^ in Area 1 and 1.383 ± 0.41 mg m^−2^ d^−1^ in Area 2. The difference in mean values was not statistically significant.

When compared the values obtained for the gas production rates obtained in this study with the rates obtained in studies carried out in different environments and countries, can observe that our rates showed lower values (Table 3). According to the table, the lowest values of methane production were observed in studies that use such methodology measurement of accumulation of gas in the head space.

**Table 3:** **Rates of CH_4_ and CO_2_ production in soils intermittent or permanently inundated**.

**Site/Country**	**CH_4_ production (mg m^−2^ d^−1^)**	**CO_2_ production (mg m^−2^ d^−1^)**	**Method**	**References**
Rice field 1/Rio Grande do Sul, Brazil	0.04 ± 0.02	1.18 ± 0.3	Measured by accumulation of gas in the head space	This study
Rice field 2/Rio Grande do Sul, Brazil	0.02 ± 0.03	1.38 ± 0.41		
Rice field (bare soil)/Punjab, India	1.68 to 49.44	–	Measured by closed chamber technique	Khosa et al., 2010
Rice field (transplanting of rice crop)/Punjab, India	0.96 to 22.32	–		
Rice field 1/Gujarat, India	2536 to 17295	–	Measured by static chamber technique	Kumar and Viyol, 2009
Rice field 2/Gujarat, India	4838 to 10342	–		
Swamp/USA	83 to 155	–	Measured by chamber technique	Wilson et al., 1989
Marsh/USA	146 to 912	–	Measured by chamber technique	Alford et al., 1997
Permanently inundated wetland/USA	153.71	–	Measured by gas sampling chambers technique	Altor and Mitsch, 2006
Freshwater wetlands/USA				Mitsch and Gosselink, 1986
(a) Rice paddies	90.83	–	–	
(b) Michigan swamp	146.93	–	–	
(c) Dismal swamp/virgínia	1.33 to 20.03	–	–	
(d) Louisiana tidal freshwater marsh	587.75	–	–	
Lake dagow/Germany	1.40	–	Measured by accumulation of gas in the head space	Casper, 1992
Lake fuchskuhle/Germany	0.47	–		
Lake stechlin/Germany	0.32	–		

## Discussion

In this study we found higher CO_2_ production in Area 2 than Area 1, associated with higher carbon and organic-matter contents in the soil. Moreover, there was significant difference concerning sampling time, but no difference between areas. The overall CH_4_ production as well as the BA obtained from water/sediment interface sampling did not differ significantly between the areas and sampling time.

Organic-carbon content parallels the rate of methane emission in rice fields (Kumar and Viyol, 2009). Differences in the organic chemistry of a specific soil type (Yavitt and Lang, 1990) and in the composition of the organic matter between environments, whether they are more labile or more refractory, influence mineralization (CO_2_ and CH_4_ formation) in the presence or absence of oxygen (Bastviken et al., 2003), resulting in variations in methane production rates between different wetland samples (Yavitt and Lang, 1990). Soil characteristics and microbiota, associated with other factors, and could result in different responses related to availability and carbon cycling (Österreicher-Cunha et al., 2012). The observed differences in the soil composition of the two areas used in this study, related to the % carbon and organic matter content, should be result in diverse responses to gas production in the samplings carried out over the cycle of rice crops.

The structure of the soil bacterial community, although it was not evaluated, was probably important for the gas emissions observed in this study. The numbers of methanogenic and methanotrophic bacteria as well as the type of methanotrophic bacteria affect CH_4_ production (Wang et al., 2008). However, according to Krüger et al. (2005), observed variations in methanogenic processes are probably not caused by changes in the size of the methanogen community but in its activity. Observations on the pathway of CH_4_ formation showed that substrate conditions most affect the methanogen community structure and function (Chin et al., 2004). The highest rates of methane and carbon-dioxide production, as well as microbial abundance from water-sediment interface samples occurred until the third sampling, before the rice plants had attained their maximum biomass (at the end of the culture cycle) in both areas. Although a number of environmental controls over bacterial respiration have been studied in wetlands (Segers, 1998), this fact implies that, probably, the differences in soil organic matter, rather than plant growth, drive most of the microbial activity, in concordance with results obtained by Welsch and Yavitt (2007). The different effects of organic matter may be closely related to the content of easily decomposable organic matter (Hou et al., 2000), as well as the release of more-labile substrates that can be more easily assimilated by the microorganisms present in the water and sediment surface layers (Fonseca et al., 2004). Otherwise, the continuum of decomposability of the organic matter in soil can be altered by interactions with minerals within matrices capable of stabilizing potentially labile organic matter against biological oxidation (Baldock and Skjemstad, 2000), producing changes in the organic matter mineralization processes present in the soil of different areas. These variables probably influenced the methane production rates especially in Area l, even taking into account the differences in organic-matter concentrations.

The relationships between plants and soil microorganisms are important for bacterial activity (Hogberg and Read, 2006), and indirectly affect gas production. Methanotrophs are a diverse group of aerobic bacteria mainly found on the rhizosphere of aquatic plants, and their growth is favored by this aerobic microsite in the process of CH_4_ consumption (Wang et al., 2008). Increased soil carbon contents often stimulate microbial growth and activity, which can increase the availability of soil nutrients and enhance plant growth (Weihong et al., 2000). The presence and the different species of macrophytes generally reduce GHG fluxes, due to a significant impact on oxygen dynamics, mainly via their capacity to increase redox potentials and dissolved oxygen concentration (Maltais-Landry et al., 2009). According to Bouchard et al. (2007), rapid root production and increased rooting depth enhance CH_4_ oxidation to a relatively greater degree than methane production.

In this study, methane concentrations showed a decreasing gradient in successive samplings, especially in Area 1, in contrast to plant growth. The plant growth stage can affect the production of gases considering the development of underground biomass, and potential loss or GHG though leaves. The reduction in the methane concentrations observed as the cultivation cycle evolved could be an effect of plant and root development, substantially, or totally inhibiting methane production (Gilbert and Frenzel, 1998) and producing the irregular values obtained in this study. These results are in agreement with those of Picek et al. (2007), once that they found a gradual decline in both CH_4_ and CO_2_ toward the end of the growing season. The plants could attenuate methane emission, facilitating its oxidation by the transport and release of oxygen through the roots located in the aerobic surface zone of the sediment (Schrope et al., 1999; Whalen, 2005). However, measurements of growth of the rice were not performed throughout the cycle, which needs to be stated as a variable to be considered in the follow up works.

Permanently flooded areas produce more methane than those that are intermittently flooded (Kumar and Viyol, 2009), with or without the presence of emerging macrophytes (Altor and Mitsch, 2006). Variations in the water level can affect the emission of gases such as methane and CO_2_ (Ratering and Conrad, 1998; Cheng et al., 2007; Hirota et al., 2007). In this study, the first sampling conducted in Area 2 resulted in the lowest methane production level of the entire cultivation cycle, when the areas reached minimal water layers. The drainage of the rice paddies resulted in decreased methane production, since this would increase the penetration of oxygen into the soil and suppress the production of this gas. On the other hand, the aeration process can result in an increase of CO_2_ production, since that the degradation of organic matter is more efficient under oxidized conditions (Ratering and Conrad, 1998). Oxygen penetration into the soil permits oxidation of reduced sulfur to sulfate and ferrous iron to ferric iron. Sulfate and iron (III) favor the action of sulfur- and iron-reducing bacteria that use acetate and hydrogen substrates more efficiently than methanogenic bacteria (Ratering and Conrad, 1998). These processes lead to competition for the substrates, as observed by Minello (2004) in a study conducted on lagoon sediment, and, consequently, the hydrogen and acetate concentrations drop to levels that prohibit methane production (Ratering and Conrad, 1998; Conrad, 2002).

Under anaerobic conditions, the mineralization of organic carbon to CO_2_ and CH_4_ is carried out by a *consortium* of microbes, where the complex organic polymers are initially degraded by fermenting bacteria to yield a few simple products, which are subsequently used by methanogens to produce CH_4_ (Conrad, 1989). The relative proportion in which CO_2_ and methane gases are produced from organic material depends on the presence of sufficient inorganic oxidants, such as nitrate, manganese (IV), iron (III) and sulfate. When this is the case, the organic material is principally degraded to CO_2_, increasing its production, and relatively little methane is produced (Yao and Conrad, 2000; Krüger et al., 2001). Our results showed that the mean rates of CO_2_ production were higher than those of CH_4_ in both areas. These results are also in agreement with those of Picek et al. (2007), who found that only 10% of the total carbon emissions from a constructed wetland were in the form of CH_4_; and with Bastviken et al. (2003), who measured the mineralization of organic carbon in oxic and anoxic lake sediments and found that carbon-dioxide production dominated that of methane as a mineralization product. Also, the lower temperatures used in the incubations, closer to the value that is best suited for CO_2_ production, as well as variables such as the products originating from the breakdown of organic matter, increase the likelihood of more efficient use of the exudates released by the organic matter throughout cultivation (Silvola et al., 1996) and explain the higher production of CO_2_ than methane, as seen in the results obtained in the two cultivated areas.

The combination of low soil pH and potential methanotrophy, together with the addition of fertilizer, may have caused a low methane production in this temporary wetland. During the crop cycle of the rice paddies, two applications of urea fertilizer were made in both areas, during the initial phase of cultivation. Whereas Krüger and Frenzel (2003) observed little to no change in overall CH_4_ emissions from rice fields in response to increased N-fertilization, other studies have found variable effects. The application of fertilizers could lead to a reduction rather than an increase in methane emission in wetlands formed by rice fields (Bodelier et al., 2000a). Urea increases soil pH and reduces methane production, especially when it is incorporated at some depth, since the majority of methane-producing organisms are neutrophilic and their production slows at pH values lower than 6.4 and higher than 7.8 (Wang et al., 1992). Also, the effect of fertilization stimulates the activity of methanotrophic organisms, controlling the emission of methane close to the plants, thus reducing its emission (Bodelier et al., 2000b).

The present study shows lower values compared with the data obtained in studies performed in other ecosystems, such as rice fields and wetlands of the Northern hemisphere. The lowest values of methane production were observed in studies that use such methodology measurement of accumulation of gas in the head space. However, comparison of rates of methane production from different studies is very difficult, since many variables such as environmental factors need to be considered in the analysis of gas production rates. In addition to these factors, different methods are used and the rates are dependent on both temperature and hydroperiod (Mitsch and Gosselink, 1986), which may result in variations in the data, hindering the analysis and interpretation of results.

The results obtained in this study suggest that the carbon and organic-matter contents in the soil of irrigated rice cultivation areas may have been used in different ways by soil microorganisms, leading to variations in CH_4_ and CO_2_ production. Identifying the conditions in which these processes occur and the types of organisms involved would help explain the variation observed in this and other studies. Our data suggest that differences between ecosystems, such as the carbon and organic matter content presents in temporary wetlands soil, could result in variations of CH_4_ and CO_2_ production rates even for limited periods of time.

### Conflict of interest statement

The authors declare that the research was conducted in the absence of any commercial or financial relationships that could be construed as a potential conflict of interest.
